# Timing of the Sense of Volition in Patients With Schizophrenia

**DOI:** 10.3389/fnins.2020.574472

**Published:** 2020-10-30

**Authors:** Sarah Pirio Richardson, Antonio I. Triggiani, Masao Matsuhashi, Valerie Voon, Elizabeth Peckham, Fatta Nahab, Zoltan Mari, Mark Hallett

**Affiliations:** ^1^Human Motor Control Section, National Institute of Neurological Disorders and Stroke, National Institutes of Health, Bethesda, MD, United States; ^2^Department of Neurology, University of New Mexico Health Sciences Center, Albuquerque, NM, United States; ^3^Human Brain Research Center, Graduate School of Medicine, Kyoto University, Kyoto, Japan; ^4^Department of Psychiatry, University of Cambridge, Cambridge, United Kingdom; ^5^Central Texas Neurology Consultants, Round Rock, TX, United States; ^6^Department of Neurosciences, Movement Disorder Center, University of California, San Diego, San Diego, CA, United States; ^7^Cleveland Clinic Lou Ruvo Center for Brain Health, Las Vegas, NV, United States

**Keywords:** schizophrenia, agency, passivity phenomena, Bereitschaftspotential, Libet’s clock, readiness potential, BP

## Abstract

Schizophrenic patients often do not have the sense that they direct their own movements or author their own thoughts (passivity phenomena). As willing must precede movement to be causal and thus generate the sense of agency, it is possible that the timing between the senses of willing and movement is shortened in schizophrenia. We tested the subjective perception of this time interval in patients with schizophrenia using a method based on Libet’s paradigm, in which subjects specify a time W – the time of willing a movement – and a time M – the time that movement occurred. Patients with schizophrenia and healthy volunteers made voluntary movements at times of their own choice while looking at a fast-rotating clock on a computer screen and reported when their movements were willed and made. We recorded surface electromyography to determine the time of actual movement, and electroencephalography to record brain potentials associated with movement. Results showed a significantly reduced interval between the reported M and W in patients with respect to the healthy volunteers (*p* < 0.05). Specifically, patients did not report a significant difference in the timing of W at 19 ms prior to movement onset and M at 7.4 ms prior to movement onset (*p* > 0.05), while the control group experienced a time W at 100 ms prior to movement onset and this differed significantly from their time M at 19 ms prior to movement onset (*p* < 0.01). These results suggest that patients with schizophrenia do have an altered timing of awareness of action – or an impaired judgment of the sequence of events – and that this might be etiologic in the development of the abnormal sense of agency.

## Introduction

Libet et al. (1983) used a fast-rotating clock to demonstrate in healthy subjects that the perception of having willed voluntary movement was preceded by cortical activity measurable by electroencephalography (EEG). Their subjects identified on the clock at what time they decided to move (time W), and when they had the sense that they moved (time M). The EEG measure was the Bereitschaftspotential (BP), a long, slow EEG negativity over the vertex that begins about 1 s before movement. They concluded from these results that the sense of deciding to act may not actually represent a conscious decision, but merely be a conscious awareness arising in the middle of an unconscious process that precedes movement.

Since the Libet et al. (1983) experiment, many criticisms have been raised against his timeline of voluntary movement. For example, Haggard et al. (1999) proposed the problem of “prior entry bias,” in which events that require more attention are perceived as happening earlier. Gomes (2002) found fault with the requirement to delineate W and M – phenomena which he finds to be indistinguishable as most people have a “unitary awareness” of voluntarily moving.

These objections highlight the need for further studies into the questions raised by Libet et al. (1983) initial experiment. Many movements, such as walking, are executed and even corrected without awareness. The driving interest behind this research is then not only why the mind should be “behind” the brain in awareness of movement, but why awareness is necessary at all. Schizophrenia represents a unique case against “unitary awareness,” as many patients with schizophrenia have one component of this awareness (the awareness of movement) without the other (the awareness of deciding to move, or at the least, misattributing the source agent of W) (Graham et al., 2014).

Schizophrenia comprises a heterogeneous group of mental disorders characterized by disturbances in form and content of thought, altered mood, and impaired perspective of self and external environment and includes symptoms like passivity (Herbener and Harrow, 2019). Passivity symptoms demonstrate a crucial, subjective change in how self is experienced and allows for an external agent to substitute for the self in generating thoughts, sounds, and movements (Gallagher, 2004; Graham-Schmidt et al., 2018). Passivity phenomena can then act as a model for understanding how the normal conjoining of a motor activity with the intention to move can unravel and leave a person susceptible to delusions of external control. Moreover, passivity was found related to the dysfunction of visuomotor action monitoring, suggesting that psychotic passivity experiences might result from abnormal central action monitoring mechanisms (Schnell et al., 2008). Another possible role would be played by the corollary discharge (CD), a feedforward mechanism that normally contributes to the emergence of the sense of agency, and that appears to be altered in schizophrenia (Poletti et al., 2019). A study found that the microstructural integrity in the pathway connecting frontal eye fields (FEFs) with the mediodorsal thalamus (MD) was compromised in patients affected by schizophrenia. This was seen related to an oculomotor CD dysfunction and the severity of psychotic symptoms (Yao et al., 2019).

Healthy people, such as in Libet et al. (1983) study, are able to identify an experience of will that precedes voluntary movement and know “I moved.” With W before M, these people have an awareness of the intention to move and can appropriately attribute causality (Wegner, 2003). The purpose of this study is to determine the timing of willing and initiating a movement in a disease state where the awareness of intention and action are impaired. We hypothesize that in schizophrenia patients, the separation between W and M will be shorter than in healthy volunteers, resulting in an altered sense of causality and hence conscious will. Moreover, we hypothesize that this altered perception of W is related to the presence and the severity of passivity phenomena.

## Materials and Methods

### Study Participants

Fifteen patients with DSM-IV diagnosis of schizophrenia (American Psychiatric and Association, 1994) who had volunteered to participate in the clinical research unit of the Clinical Brain Disorders Branch at the National Institute of Mental Health (NIMH) were enrolled. One patient withdrew from the study due to change in health status. The enrolled patients were 4 women and 10 men (mean age 28.9 years, range from 19 to 57 years). Fifteen healthy, age-matched volunteers were recruited; one withdrew due to change in health status leaving six women and eight men (mean age 31.3 years, range from 18 to 57 years). One subject decided to withdraw after signing the consent but before participating in the study. All patients were under second-generation antipsychotic treatment, as required by the National Institute of Neurological Disorders and Stroke Institutional Review Board who approved this study. All patients were clinically stable at the time of testing. Patients were asked to give informed consent. Subjects were able to demonstrate that they understood, among other factors, that participation was voluntary, that it would not benefit them, and that they could withdraw from the study at any time. Patients also demonstrated that they understood that not participating was an option. As part of documenting informed consent, patients were asked to take a written test covering details of the protocol and its benefits and risks. Patients who were unable to score at least 75% on the written test were excluded.

A neurologist performed the history and physical examination for schizophrenic patients and healthy volunteers. Patients on the clinical research unit had to meet rigorous criteria in order to participate in research. Exclusionary criteria included history of traumatic brain injury, known comorbid neurological disorders, including epilepsy, history of drug and alcohol abuse. A psychiatrist administered clinical rating scales [i.e., appropriate sections of the Structured Clinical Interview for DSM-IV (SCID)] (First et al., 2002). Moreover, since patients with schizophrenia frequently exhibit deficits in numerous neurocognitive domains, including attention, the psychiatrist administered also a comprehensive battery of neuropsychological tests to determine whether the patients were able to participate the study. This battery included Wechsler Memory Scale-Revised (Wechsler, 1987), Wechsler Adult Intelligence Scale-Revised (Wechsler, 1981), Trail Making Test A and B (Tombaugh, 2004), and the vigilance and distractibility versions of the continuous performance task (CPT) (Gordon and Mettelman, 1987).

### Times S, M, W

**FIGURE 1:**
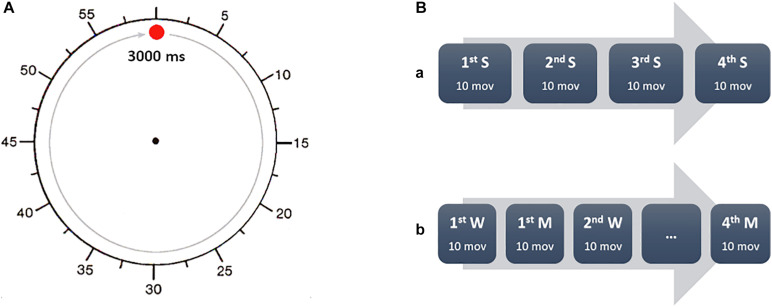
Experimental task sequence. **(A)** During every task, the participants were asked to fix the center of the clock (replica of clock used in the study). The fast-moving red dot rotated clockwise around the perimeter of the clock with a full revolution accomplished in 3 s. The time was reported as for a common clock, so every 5 s mark was actually 250 ms. **(B)** The participants were asked to report the time (S) of external electrical stimulation (A) in a separate session, before performing the task with the W and M conditions. W and M were alternated in four blocks of 10 movements each. The participants were asked to report every time aloud, after every trial.

All participants watched a training video on the tasks. The researchers followed a standardized script when giving task instructions. The healthy subjects and schizophrenic patients watched a clock presented on a computer screen. The clock was a circle with a red dot, which revolved around the clockface in 3 s. A circular scale was marked around the periphery of the clock, with tick marks at each “5 s” position, actually marking 250 ms of real time (Figure 1). There were three sessions, S, W, and M. During the session S, subjects received a short somatosensory stimulus; during the session W and M they had to move spontaneously. The computer with the clock recorded the time somatosensory stimuli were given, as well as the electromyographic (EMG) onset of movement.

Time S was measured by sessions in which subjects watched the clock and noted the time at which they perceived a short somatosensory stimulus (time S), repeated 40 times. The stimulus was a non-painful electrical shock of an intensity set at twice the personal sensory perception threshold. The magnitude of the non-painful stimulation was established by a series of increasing stimulus intensities to determine the subjective sensory threshold. This evaluation was performed during a separate session before the proper experiment. The stimulus consisted of a 2 ms pulse, delivered by a ring electrode. In one of the analyses, the sensory stimulation trials were used to adjust the interpretation of times W and M. Moreover, it was a useful control for the ability of the subject to report with a minimum accuracy the time of an objective movement. By Libet et al. (1983) procedure, we subtracted S from W and M before reporting these times as results.

Times W and M were measured in sessions in which subjects were asked to make a voluntary movement with the right arm approximately every 5–6 turns of the clock. The subjects voluntarily made a brisk movement of wrist and finger extension, which was relatively stereotyped, rapid and short in duration. During this session subjects alternated between blocks of noting the time at which they had the conscious intention to move, W (or felt that some external force ordered their movement), and the time at which they felt the initiation of movement M. The blocks consisted of 10 movements each, alternating between W and M as the reported time. Four blocks each of W and M were studied in total. Subjects were asked to note the time of W, M, and S and then wait until a few rotations of the clock have passed before saying the time aloud. This will prevent the presence of artifacts (due to head movements or speech) that would have affected the electrophysiological recordings. Furthermore, following Libet’s procedure, every participant was instructed to keep their gaze on the center of the clock, even when they had to note the position of the hand of the clock, to avoid eye movements that could have produced ocular electrical artifacts. Finally, again in accordance with Libet’s original experiment, the subjects were free to blink when they had the urge to so, but were instructed, after a blink, to wait for at least another revolution of the clock before moving.

### Electroencephalography and Electromyography

EEG was used to record brain electrical potentials throughout the experiment. A standard EEG electrode cap, with 28 electrodes placed according to the international 10–20 system, was used and placed using standard measurements. Impedance was kept at less than 5 kOhm. The EEG was filtered with a DC-200 Hz low-pass filter and digitized at a rate of 1000 Hz. The EMG signal was recorded from disposable surface silver-silver chloride electrodes over the right extensor digitorum communis in a bipolar montage. The EMG signal was rectified, integrated, and fed to a homemade Schmidt trigger that was set to trigger at the EMG burst onset. An electro-oculogram (EOG) was recorded in all subjects. Each session was stored in digital format for off-line analysis.

### EEG and EMG Analysis

For all the EEG and EMG analyses we followed the methods delineated by Karp et al. (1996). Recordings were visually inspected off-line. Artifacts and insufficiently brisk movements were excluded from analysis. The EOG recording was used to verify the ocular conditions in every trial, detecting the epochs affected by blinks, saccades, and generic eye movements. During W and M trials, epochs were marked and segregated according to whether the subject was reporting W or M. Data were epoched accordingly to the rectified EMG burst onset, considered the trigger for the movement. Specifically, every epoch had a duration of 4 s, starting 3500 ms before trigger, until 4500 ms after the trigger. Since the premotor potential onset is defined by a rise in negativity above baseline (established as the mean amplitude from approximately −3500 to −3000 ms before EMG onset) in the averaged tracings, the amplitude of every potential was referred to that baseline activity. The voltage analysis was focused on the movement-related cortical potentials (MRCPs). In particular, the MRCP on the vertex (electrode Cz) was divided into the classical two components: the Bereitschaftspotential 1 (BP1), the earlier widespread component, and the Bereitschaftspotential 2 (BP2), the later steeper component. This was done for both the W and M trials.

### Positive Symptoms

The scale for assessment of positive symptoms (SAPS) (Andreasen, 1984) was used to assess the severity and presence of psychotic symptoms in 12 out of the 14 patients. Two patients declined the assessment. The passivity symptoms were derived by the following items of the SAPS, belonging to the Delusions domain: 15, delusions of being controlled; 16, delusions of mind reading; 17, thought broadcasting; 18, thought insertion; and 19, thought withdrawal (Frith, 2005; Schnell et al., 2008). The level range was 0 (none) to 5 (severe). The presence was considered with a score higher than 0, while for the severity the sum of the five items was considered. As a control analysis, we similarly computed the Hallucinations domain (items 1–7), and the whole Delusions domain (items 8–20).

### Statistical Analysis

Noteworthy, Benjamin Libet and colleagues did not provide any statistical analysis, and the entire work was purely descriptive. Among the most recent Libet’s replications involving patients, we found that Jungilligens et al. (2019) compared healthy volunteers with patients affected by epileptic seizures. They used a MANOVA with the factor group and the score of scales of dissociating experience and W–M difference value as dependent variables. Baek et al. (2017) used Libet’s paradigm with the fMRI. They divided patients with functional neurological disorders in two groups, with or without positive motor symptoms, compared in a simple ANOVA model with healthy volunteers. Edwards et al. (2011) for a Libet’s replication with healthy volunteers and patients with psychogenic tremor used a two-way ANOVA with the condition (M, W) and group (patients and controls) as main factors.

Regarding the concept of psychotic symptoms, Schnell et al. (2008) hypothesized a relationship between passivity symptoms and the BOLD response within the monitoring network during an object recognition task. To test it, they used a simple regression analysis only in the patient group. Graham-Schmidt et al. (2018) tested the experience of lost agency in patients with Schizophrenia, using the projected hand illusion (PHI) with active and passive movements. The analyses were performed with a linear mixed model with questionnaire responses as the dependent variable and presence of passivity symptoms (Controls, Current, Past, or Never), movement condition (Active or Passive), and delay condition (Synchronous or Asynchronous) as the fixed effects. Yao et al. (2019), using the probabilistic tractography, analyzed the integrity of the pathway projecting from the superior colliculus to the FEFs, via the MD. This pathway conveys oculomotor CD associated with saccadic eye movements in non-human primates. They used the Spearman correlation between the measure of the integrity of the path and the psychotic symptoms. In line with the previous experiments, we decided to use a linear model and a simple regression for the symptoms and the reported times only for the patients. Moreover, we used the S condition to correct the reported times, as follows.

W and M times were compared using a mixed model where the measures for M and W (Condition) represented the repeated factor and schizophrenia patients versus healthy volunteers were the grouping (Group) factor. The subjects were used as the random factor. We also included an evaluation of the Bayes factor (BF), as a complementary indicator to the classical hypothesis testing. This analysis was performed adjusted for S. *Post hoc* testing was performed with pairwise comparisons of estimated marginal means (EMMs) with Tukey adjustment. *p*-Values less than 0.05 were considered significant. The same model was used to analyze the differences in the onset of the MRCPs (i.e., BP1 and BP2) and the amplitudes at the time 0 (onset of EMG). Depending on the normality of data, we performed Pearson’s or Spearman’s rank correlation to look at relationship between passivity and psychotic symptoms in schizophrenia.

For all the statistical analysis, we used R (version 4.0.2, R Core Team, 2018) and GraphPad Prism (version 8.4.0 GraphPad Software, LLC). The normality of the variable was assessed using the Shapiro–Wilk test. ROUT test (Q = 1%, Motulsky and Brown, 2006) was used to reveal the presence of eventual outliers.

## Results

### Differences in Times M and W

**FIGURE 2:**
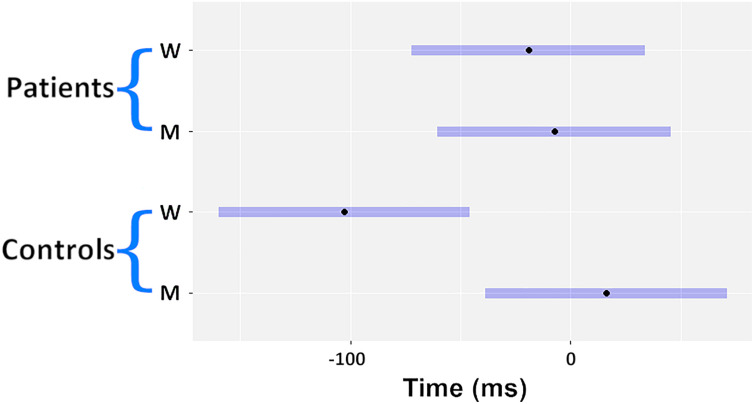
Dot and whiskers plot of the estimated marginal means (EMM).

Reported times were normally distributed. For the S session (normality *p* = 0.5 for the patients, *p* = 0.1 for the volunteers) patients with schizophrenia reported feeling the sensory stimuli at 59.4 ms (SEM 12 ms) after the stimuli were applied. Healthy volunteers reported time S at 11.7 ms (SEM 14.2 ms) after stimuli onset. This difference was significant (*p* = 0.02). Time S was then used to adjust time W and M on an individual basis as discussed in the Methods. In Table 1 the three values were reported as mean and standard error of the mean for both the groups (see the Supplementary File for the individual values). ROUT test revealed the presence of one outlier, which was removed from the statistical model. The mixed model with the reported values of M and W (adjusted for S) was significant for the Report type factor (df = 24, *F* = 7.6, *p* = 0.011, BF = 2.9) and for the interaction Report type x Group (df = 24, *F* = 5.9, *p* = 0.02, BF = 2.9). The BF related to the non-significant factors were smaller than 1. *Post hoc* comparisons showed that patients with schizophrenia did not report a significant difference in the timing of M and W (11.7 ms) (SEM 32.9, *p* = 0.98, effect size = 0.12). Healthy volunteers by contrast did show a difference between M and W (128.8 ms) (SEM 35.1, *p* = 0.006, effect size = 1.36). In Figure 2 the marginal means are depicted in a dot and whiskers plot.

**TABLE 1:** Results of Times S, M, and W expressed as mean, standard error of the mean by group and *t*-test results as *p*-values.

	Schizophrenia patients mean^ (SEM)	Healthy volunteers mean^ (SEM)	*p*-Value
Time S	59.4 (12)	11.7 (14.2)	0.016
Time M*	−7.5 (21.6)	28.1 (20.8)	0.7844
Time W*	−19.1 (33)	−100.8 (25.5)	0.0068

^Mean times shown in milliseconds. *Times adjusted for Time S.

As a control analysis, within the schizophrenia group, patients were asked after the experiment if they “felt a difference” between Time M and Time W. Four of the patients responded “yes” and 10 responded “no.” For the subjects who felt a difference, their time W was −151 ms (SEM 51.6) and M was −51 ms (SEM 35.6). In contrast the subjects who did not feel a difference, time M and W were 33.6 ms (SEM 27.8) and 10 ms (SEM 25.6), respectively. In the subgroup analysis, there was a significant difference between patients who felt a difference in W and those who did not (*p* = 0.0026).

### Positive Symptoms

The mean total score for the SAPS was 19.1 (5.28 SEM). The presence of passivity symptoms was found in six patients, and the mean score was 9.67 (1.6 SEM). Five patients did not have passivity symptoms. The mean total score for the hallucinations was 5.50 (1.35 SEM), while the mean total score of hallucinations plus delusions was 16 (3.9 SEM). There was no correlation between the subjective feeling of a difference between W and M trials and the severity of passivity symptoms. There were no correlations found when taking into account the presence of hallucination symptoms alone (mean 5.50, SEM 4.7) or with hallucinations symptoms together with the delusion symptoms (Table 2).

**TABLE 2:** Correlation analysis of SAPS with times M and W.

SAPS items	Correlation with W	*p*-Value
SAPS total	−0.10	0.75
Hallucinations + Delusions	−0.20	0.53
Hallucinations	−0.18	0.58
Passivity*	0.27	0.61

*Correlation with only six subjects. ^Spearman r.

### Movement Related Cortical Potentials

**FIGURE 3:**
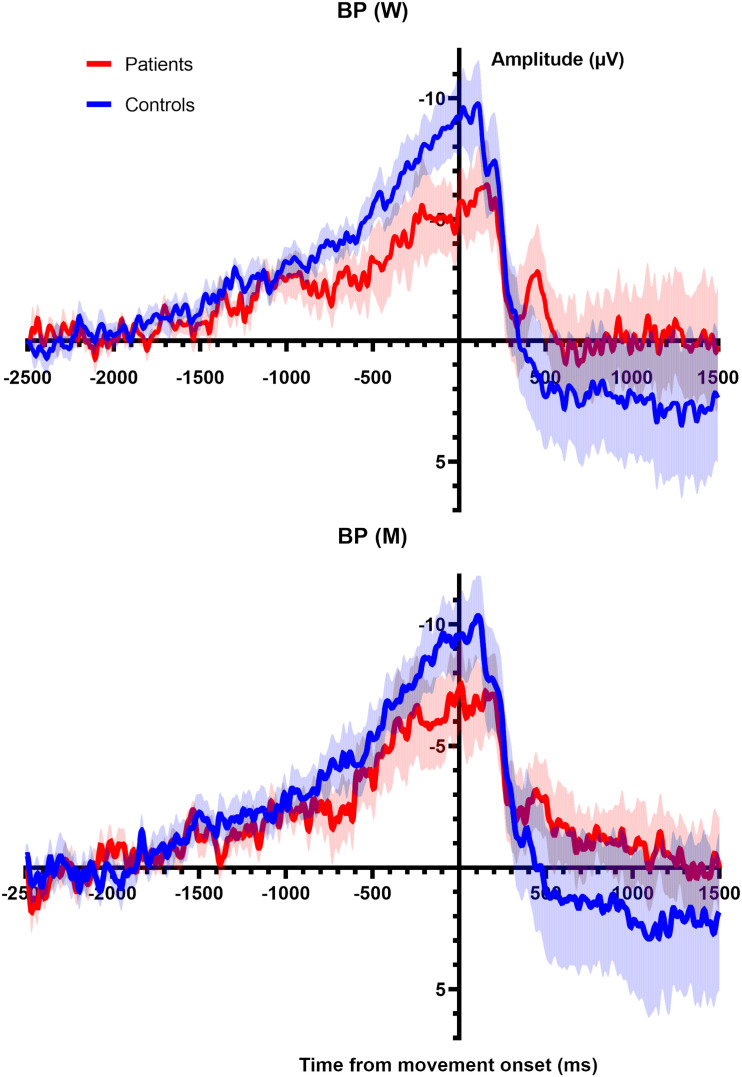
**(Top)** Averaged BP (main line) with standard error of the mean (shaded area) for all participants shown for electrode CZ for the W trials. Epoch begins 2.5 s prior to movement onset and 1.5 s after movement. **(Bottom)** Averaged BP (main line) with standard error of the mean (shaded area) for all participants shown for electrode CZ for the M trials. Epoch begins 2.5 s prior to movement onset and 1.5 s after movement.

As a control analysis we analyzed the shape and the onsets of the MRCPs in both conditions (W and M). Due to the presence of artifacts, only 11 Healthy volunteers and 10 patients were analyzed. Figure 3 depicts the average over all subjects of the MRCPs for W trials and M trials. Table 3 summarizes the values (mean and SEM) of the amplitudes of the BPs and the onset of every MRCPs (i.e., BP1 and BP2). The amplitudes were computed at time 0, in correspondence with the EMG onset. Two independent mixed models (for amplitude and onset, respectively) did not unveil any significant difference for Group, Condition or the interaction (see the Supplementary File for the individual values). Thus, no *post hoc* tests were performed.

**TABLE 3:** Onset time of BP1 and BP2 for W and M in the patients and the healthy volunteers.

Condition	Measure	Patients		Control	
W	BP1 onset	−2108.0	(−254.3)	−2159.2	(−255.5)
	BP2 onset	−422.8	(−53.2)	−546.9	(70.2)
M	BP1 onset	−2305.6	(263.3)	−1971.4	(307.3)
	BP2 onset	−672.4	(99.2)	−556.7	(66.9)
W	BP amplitude	−5.6	(−1.6)	−9.1	(−1.4)
M	BP amplitude	−7.4	(−1.9)	−9.6	(−1.6)

*Data are expressed as means (standard error).*

## Discussion

We confirmed the findings of Libet et al. (1983) that in healthy volunteers, time W follows the onset of the BP but precedes time M. In contrast, patients with schizophrenia had a similar onset of BP as healthy volunteers but in most there was no distinct perception of W preceding M. Importantly, the unconscious generation of voluntary movement was indistinguishable between the groups based on the BP analysis, but the conscious perception of movement generation was clearly different between the groups. The experience of moving and willing almost merged in the patient group. These results suggest that patients with schizophrenia *do* have an altered timing of awareness of action—or an impaired judgment of the sequence of events—compared to healthy individuals. This echoes results found in patients with functional (psychogenic) tremor and an altered sense of agency where W was shifted later and M and W were indistinguishable (Edwards et al., 2011). A similar result was found in subjects affected by dissociative seizures, brief episodes of disrupted awareness and behavioral control, who showed a very short difference between the reported timing of M and W (Jungilligens et al., 2019). Another Libet-like experiment, using the fMRI, found that also patients with mixed functional neurological disorder showed a reduced difference between the reported M and W (Baek et al., 2017).

Patients with schizophrenia have shown “hyperbinding” or “hyperassociation” in the conscious experience of events separated in time, meaning that patients over-associated their voluntary actions with a sensory cue (Haggard et al., 2003). In that experiment, patients “over-attributed” their agency (Haggard et al., 2003). One interpretation of our results is that schizophrenic patients do have a difference between M and W just as healthy volunteers, but due to excessive binding, those events are indistinguishable and reported similarly in time. This would suggest that agency persists but that the process of *willing* a movement and *moving* are the same—not that the movement was under alien control or other such phenomena. Alternatively, it is possible that they are not the same events but so much closer in time that a distinction is difficult to detect with this methodology. This is consistent with other work in patients with schizophrenia, which showed patients tend to underestimate a temporal interval between events (Franck et al., 2005).

After the task, patients were asked if they “felt a difference” between W and M, and four patients reported that they did. They performed similarly to healthy volunteers with time W reported at 151 ms prior to movement onset. There was no relationship between their passivity scores or other disease measures to account for this difference in experience—and performance on the task. Of note, all patients were on second-generation antipsychotic medications at the time of the task, which may have affected the passivity rating scales. It is possible that the scales used were not adequately sensitive to detect this change. In addition, the analysis was not powered to detect changes between individual patient performance—only to look at patients and healthy volunteers in a group fashion.

One of the important facets of Libet et al. (1983) original work was the presence of the Bereitschaftspotential preceding the report of time W. He interpreted this phenomenon as a temporal progression of movement beginning with subconscious preparation for movement followed by conscious awareness of the intention to move—quite proximal to the movement itself. Other investigators have found BP1 amplitudes reduced and prolonged latency of BP1 in schizophrenic patients compared to healthy volunteers (Dreher et al., 1999; Northoff et al., 2000). We did not find significant differences in latency or in amplitude between patients and healthy volunteers. There are certainly differences in study methodology that may account for the disparate results. In the previous studies, the patients were asked to make a self-paced movement of the finger every 4–5 s and fixate their gaze in the middle of a computer screen as opposed to our study where patients had their focus clearly on the clock and had to respond to questions of timing. This raises the question of “attention” versus “intention” in interpreting this work.

Subjects in this experiment were asked after an event occurred to reconstruct the timing of their intention. Work by Lau et al. (2004) showed that brain activity differed depending on whether the subject attended to the W task or the M task. In the W task, there was increased blood flow in dorsal prefrontal cortex, intraparietal sulcus and the pre-supplementary motor area (SMA). They suggested that the pre-SMA activation could be the reflection of intention; other work suggests that the parietal cortex is important as well (Sirigu et al., 1999). In any case, the question of exactly what the subjects are attending to during the “intention” conditions is an open one (Eagleman, 2004). In all of these experiments including ours, subjects are asked to “access” this awareness of movement—if one is not asked, does one still feel this awareness (Eagleman, 2004)? In a study by Matsuhashi and Hallett, they developed methodology to measure a time T, which was not dependent on subjective report of timing—but was thought to reflect the timing of the conscious intention to move (Matsuhashi and Hallett, 2008). They, as in our current work, show that time T was found after the brain had already begun unconscious preparations for movement (Matsuhashi and Hallett, 2008). This is again consistent with a generalized preparation for movement that begins unconsciously and then progresses to a conscious awareness of intention. Whether this intention is generated prior to movement solely or modified after the movement due to a reconstruction of awareness, is not entirely clear.

An important point of view in the interpretation of our results considers the relationship between W and the defect in the forward model of movement that the brain receives as the motor signal is generated (Frith et al., 2000). One model for motor control holds that movements are guided by mental representations made before the action begins (Jordan, 1996). By this model, a motor command includes information on both the current limb position and desired limb position; the subtraction of these two states (the forward model) requires the combination of known movements. Tracing a path that our mind has already made allows us to correct for errors quickly, integrate external sensory input into the movement program, and learn from mental practice (Frith et al., 2000). Frith provided a model according to which a single cognitive mechanism underlies the symptoms that characterize schizophrenia: an impairment in self-monitoring, or failure in meta-representation of one’s own or others’ internal states and beliefs. Such impairments might be involved in a disruption of the internal model and the efferent copy of motor programs, which would lead to a lack of awareness of the intended action. Such a disruption may also be behind the experience of delusions of control in which one’s actions are experienced as if controlled by alien agents or forces. Thus, even if schizophrenics can make normal, coordinated movement, they demonstrate less of the benefits predicted by the forward model: schizophrenics have impaired central error correction and more difficulty integrating unexpected interference during acts (Frith et al., 2000). Moreover, Maruff et al. (2003) found that the abnormal forward modeling in schizophrenics with passivity included impaired motor imagery: patients often failed to account for environmental restraints when making imagined voluntary movements.

More recently, some studies focused on two distinct forward mechanisms: the integral forward model, and the auxiliary forward model (Pickering and Clark, 2014). In the former, perception involves the use of a forward model for the action. In the latter, the prediction mechanism is implemented by auxiliary circuits, like the CD, a copy of the motor command used by the central nervous system to evaluate the sensory consequences of the actions, and it is this that seems altered in schizophrenia (Poletti et al., 2019). This approach has been used to explain the symptoms in schizophrenia: the generation of the forward model is abnormal. Thus, the prediction of the sensory consequences of self-generated stimuli is inaccurate and, sometimes, even attributed to an external source (Wilkinson, 2015). We did not find any relationship between the performance and either passivity or SAPS. The limited number of patients with passivity symptoms might partially account for this result but it may be hypothesized that the abnormalities in the proposed forward model are more related to the general presence of positive symptoms, specific of the disorder, more than their severity.

In this current work, we show that patients with schizophrenia differ from healthy volunteers in their experience of the *will* to move. This altered time sequence may generate an abnormal experience of causality and, even, an abnormal experience of conscious will itself – illusion or not (Wegner, 2003).

## Data Availability Statement

The raw data supporting the conclusions of this article will be made available by the authors, without undue reservation.

## Ethics Statement

The studies involving human participants were reviewed and approved by National Institute of Neurological Disorders and Stroke Institutional Review Board. The patients/participants provided their written informed consent to participate in this study.

## Author Contributions

SP: concept and design, data analysis, data interpretation, and first version of manuscript. AT: data analysis and manuscript revision. MM: concept and data interpretation. VV: enrollments, data interpretation, and manuscript revision. EP: data recordings and manuscript revision. FN and ZM: data recordings, data analysis, and manuscript revision. MH: concept and design, data interpretation, and manuscript revision. All authors contributed to the article and approved the submitted version.

## Conflict of Interest

The authors declare that the research was conducted in the absence of any commercial or financial relationships that could be construed as a potential conflict of interest. The handling editor declared a past co-authorship with one of the authors EP.

## References

[B1] American Psychiatric and Association (1994). *Diagnostic and Statistical Manual of Mental Disorders (DSM-IV)*, 4th Edn Washington, DC: APA.

[B2] Andreasen N. C. (1984). *The Scale for the Assessment of Positive Symptoms (SAPS).* Iowa City: U.O.I. Press.

[B3] Baek K. Doñamayor N. Morris L. S. Strelchuk D. Mitchell S. Mikheenko Y. (2017). Impaired awareness of motor intention in functional neurological disorder: implications for voluntary and functional movement. *Psychol. Med.* 47 1624–1636. 10.1017/s0033291717000071 28183377 PMC5964459

[B4] Dreher J. C. Trapp W. Banquet J. P. Keil M. Gunther W. Burnod Y. (1999). Planning dysfunction in schizophrenia: impairment of potentials preceding fixed/free and single/sequence of self-initiated finger movements. *Exp. Brain Res.* 124 200–214. 10.1007/s002210050615 9928843

[B5] Eagleman D. M. (2004). Neuroscience. The where and when of intention. *Science* 303 1144–1146. 10.1126/science.1095331 14976300

[B6] Edwards M. J. Moretto G. Schwingenschuh P. Katschnig P. Bhatia K. P. Haggard P. (2011). Abnormal sense of intention preceding voluntary movement in patients with psychogenic tremor. *Neuropsychologia* 49 2791–2793. 10.1016/j.neuropsychologia.2011.05.021 21683724

[B7] First M. Spitzer R. Gibbon M. Williams J. (2002). *Structured Clinical Interview for DSM–IV–TR Axis I Disorders, Research Version, Patient Edition. (SCID-I/P).* New York, NY: Biometrics Research, New York State Psychiatric Institute.

[B8] Franck N. Posada A. Pichon S. Haggard P. (2005). Altered subjective time of events in schizophrenia. *J. Nerv. Ment. Dis.* 193 350–353. 10.1097/01.nmd.0000161699.76032.09 15870620

[B9] Frith C. (2005). The neural basis of hallucinations and delusions. *C. R. Biol.* 328 169–175. 10.1016/j.crvi.2004.10.012 15771003

[B10] Frith C. D. Blakemore S.-J. Wolpert D. M. (2000). Explaining the symptoms of schizophrenia: abnormalities in the awareness of action. *Brain Res. Rev.* 31 357–363. 10.1016/s0165-0173(99)00052-1 10719163

[B11] Gallagher S. (2004). Neurocognitive models of schizophrenia: a neurophenomenological critique. *Psychopathology* 37 8–19. 10.1159/000077014 14988645

[B12] Gomes G. (2002). Problems in the timing of conscious experience. *Conscious Cogn.* 11 191–197. 10.1006/ccog.2002.0550 12191936

[B13] Gordon M. McClure F. D. Aylward G. P. (2002). *The Gordon Diagnostic System Interpretive Guide*, 3rd Edn DeWitt, NY: GSI Publications.

[B14] Gordon M. Mettelman B. (1987). *Technical Guide to the Gordon Diagnostic System (GDS).* DeWitt, NY: Gordon Systems.

[B15] Graham K. T. Martin-Iverson M. T. Holmes N. P. Jablensky A. Waters F. (2014). Deficits in agency in schizophrenia, and additional deficits in body image, body schema, and internal timing, in passivity symptoms. *Front. Psychiatry* 5:126. 10.3389/fpsyt.2014.00126 25309460 PMC4159976

[B16] Graham-Schmidt K. T. Martin-Iverson M. T. Waters F. A. V. (2018). Self- and other-agency in people with passivity (first rank) symptoms in schizophrenia. *Schizophr. Res.* 192 75–81. 10.1016/j.schres.2017.04.024 28416095

[B17] Haggard P. Eimer M. (1999). On the relation between brain potentials and the awareness of voluntary movements. *Exp. Brain Res.* 126 128–133. 10.1007/s002210050722 10333013

[B18] Haggard P. Martin F. Taylor-Clarke M. Jeannerod M. Franck N. (2003). Awareness of action in schizophrenia. *Neuroreport* 14 1081–1085. 10.1097/01.wnr.0000073684.00308.c0 12802207

[B19] Haggard P. Newman C. Magno E. (1999). On the perceived time of voluntary actions. *Br. J. Psychol.* 90 291–303. 10.1348/000712699161413 10363348

[B20] Herbener E. S. Harrow M. (2019). Course and symptom and functional correlates of passivity symptoms in schizophrenia: an 18-year multi-follow-up longitudinal study. *Psychol. Med.* 16 1–8. 10.1017/s0033291719003428 31839019

[B21] Jordan M. I. (1996). “Computational aspects of motor control and motor learning,” in *Handbook of Perception and Action: Motor Skills*, eds Heuer E. H. Keele E. S. (New York, NY: Academic Press). 10.4324/9781315185613-1

[B22] Jungilligens J. Wellmer J. Schlegel U. Kessler H. Axmacher N. Popkirov S. (2019). Impaired emotional and behavioural awareness and control in patients with dissociative seizures. *Psychol. Med.* 18 1–9. 10.1017/s0033291719002861 31625504

[B23] Karp B. I. Parter S. Toro C. Hallett M. (1996). Simple motor tics may be preceded by a premotor potential. *J. Neurol Neurosurg. Psychiatry* 61 103–106. 10.1136/jnnp.61.1.103 8676135 PMC486469

[B24] Lau H. C. Rogers R. D. Haggard P. Passingham R. E. (2004). Attention to intention. *Science* 303 1208–1210. 10.1126/science.1090973 14976320

[B25] Libet B. Gleason C. A. Wright E. W. Pearl D. K. (1983). Time of conscious intention to act in relation to onset of cerebral activity (readiness-potential): the unconscious initiation of a freely voluntary act. *Brain* 106 623–642. 10.1093/brain/106.3.623 6640273

[B26] Maruff P. Wilson P. Currie J. (2003). Abnormalities of motor imagery associated with somatic passivity phenomena in schizophrenia. *Schizophr. Res.* 60 229–238. 10.1016/s0920-9964(02)00214-1 12591586

[B27] Matsuhashi M. Hallett M. (2008). The timing of the conscious intention to move. *Eur. J. Neurosci.* 28 2344–2351. 10.1111/j.1460-9568.2008.06525.x 19046374 PMC4747633

[B28] Motulsky H. J. Brown R. E. (2006). Detecting outliers when fitting data with nonlinear regression – a new method based on robust nonlinear regression and the false discovery rate. *BMC Bioinformatics* 7:123. 10.1186/1471-2105-7-123 16526949 PMC1472692

[B29] Northoff G. Pfennig A. Krug M. Danos P. Leschinger A. Schwarz A. (2000). Delayed onset of late movement-related cortical potentials and abnormal response to lorazepam in catatonia. *Schizophr. Res.* 44 193–211. 10.1016/s0920-9964(99)00189-9 10962222

[B30] Pickering M. J. Clark A. (2014). Getting ahead: forward models and their place in cognitive architecture. *Trends Cogn Sci.* 18 451–456. 10.1016/j.tics.2014.05.006 24909775

[B31] Poletti M. Tortorella A. Raballo A. (2019). Impaired corollary discharge in psychosis and at-risk states: integrating neurodevelopmental, phenomenological, and clinical perspectives. *Biol. Psychiatry Cogn. Neurosci. Neuroimaging* 4 832–841. 10.1016/j.bpsc.2019.05.008 31262709

[B32] R Core Team (2018). *R: A Language and Environment for Statistical Computing.* Vienna: R Foundation for Statistical Computing.

[B33] Schnell K. Heekeren K. Daumann J. Schnell T. Schnitker R. Moller-Hartmann W. (2008). Correlation of passivity symptoms and dysfunctional visuomotor action monitoring in psychosis. *Brain* 131 2783–2797. 10.1093/brain/awn184 18713781 PMC2570714

[B34] Sirigu A. Daprati E. Pradat-Diehl P. Franck N. Jeannerod M. (1999). Perception of self-generated movement following left parietal lesion. *Brain* 122 1867–1874. 10.1093/brain/122.10.1867 10506089

[B35] Tombaugh T. N. (2004). Trail making test A and B: normative data stratified by age and education. *Arch. Clin. Neuropsychol.* 19 203–214. 10.1016/s0887-6177(03)00039-8 15010086

[B36] Wechsler D. (1981). *Wechsler Adult Intelligence Scale-Revised.* San Antonio, TX: The Psychological Corporation.

[B37] Wechsler D. (1987). *Wechsler Memory Scale-Revised Manual.* New York NY: The Psychological Corporation.

[B38] Wegner D. M. (2003). The mind’s best trick: how we experience conscious will. *Trends Cogn. Sci.* 7 65–69. 10.1016/s1364-6613(03)00002-0 12584024

[B39] Wilkinson S. (2015). Forward models and passive psychotic symptoms. *Front. Hum. Neurosci.* 9:22. 10.3389/fnhum.2015.00022 25688201 PMC4310322

[B40] Yao B. Neggers S. F. W. Rolfs M. Rosler L. Thompson I. A. Hopman H. J. (2019). Structural thalamofrontal hypoconnectivity is related to oculomotor corollary discharge dysfunction in schizophrenia. *J. Neurosci.* 39 2102–2113. 10.1523/jneurosci.1473-18.2019 30630882 PMC6507081

